# Advantages and disadvantages of 3D ultrasound of thyroid nodules including thin slice volume rendering

**DOI:** 10.1186/1756-6614-4-1

**Published:** 2011-01-07

**Authors:** Rafal Zenon Slapa, Wieslaw Stanislaw Jakubowski, Jadwiga Slowinska-Srzednicka, Kazimierz Tomasz Szopinski

**Affiliations:** 1 Department of Diagnostic Imaging, Second Faculty of Medicine with the English Division and the Physiotherapy Division, Medical University of Warsaw, ul. Kondratowicza 8, 03-242 Warsaw, Poland; 2 Department of Endocrinology, Centre for Postgraduate Medical Education, Warsaw, Poland; 3 Interdisciplinary Centre for Mathematical and Computational Modelling, Warsaw University, Warsaw, Poland

## Abstract

**Background:**

The purpose of this study was to assess the advantages and disadvantages of 3D gray-scale and power Doppler ultrasound, including thin slice volume rendering (TSVR), applied for evaluation of thyroid nodules.

**Methods:**

The retrospective evaluation by two observers of volumes of 71 thyroid nodules (55 benign, 16 cancers) was performed using a new TSVR technique. Dedicated 4D ultrasound scanner with an automatic 6-12 MHz 4D probe was used. Statistical analysis was performed with Stata v. 8.2.

**Results:**

Multiple logistic regression analysis demonstrated that independent risk factors of thyroid cancers identified by 3D ultrasound include: (a) ill-defined borders of the nodule on MPR presentation, (b) a lobulated shape of the nodule in the c-plane and (c) a density of central vessels in the nodule within the minimal or maximal ranges. Combination of features provided sensitivity 100% and specificity 60-69% for thyroid cancer.

Calcification/microcalcification-like echogenic foci on 3D ultrasound proved not to be a risk factor of thyroid cancer.

Storage of the 3D data of the whole nodules enabled subsequent evaluation of new parameters and with new rendering algorithms.

**Conclusions:**

Our results indicate that 3D ultrasound is a practical and reproducible method for the evaluation of thyroid nodules. 3D ultrasound stores volumes comprising the whole lesion or organ. Future detailed evaluations of the data are possible, looking for features that were not fully appreciated at the time of collection or applying new algorithms for volume rendering in order to gain important information. Three-dimensional ultrasound data could be included in thyroid cancer databases. Further multicenter large scale studies are warranted.

## Background

Thyroid incidentalomas are frequent and their prevalence when identified by high-resolution ultrasound is up to 67%, although most of these lesions are benign [1]. Despite the great deal of accumulated knowledge on the diagnosis and treatment of thyroid nodules, the management of thyroid carcinoma has yet to be optimized [2-6]. The recent development of three-dimensional (3D) imaging has greatly enhanced radiologic data acquisition, evaluation and storage. Ultrasound is the most useful modality for imaging thyroid nodules and it appears that 3D ultrasound could add a new dimension to thyroid cancer studies.

Three-dimensional ultrasound has been investigated for more than 20 years [7]. Due to recent developments in computer techniques and scanner technology, the acquisition of volumes with automatic three-dimensional (3D) probes has become less complicated and the quality of the images acquired by 3D ultrasound has improved to become comparable to conventional sonographic images.

There have been few reports on the examination of thyroid gland and thyroid nodule volumes by 3D ultrasound [8,9]. The presentation, size and vasculature of fetal thyroid goiter has also been evaluated with 3D ultrasound [10]. We have previously investigated the possibilities of evaluating thyroid nodules with gray-scale 3D ultrasound [11]. However, to the best of our knowledge, no previous report has described the characterization of thyroid nodules by combined gray-scale and power Doppler 3D ultrasound with evaluation of independent risk factors of thyroid cancer.

The aims of the present study were: (1) Evaluation of the feasibility and effectiveness of 3D ultrasound in differential diagnosis of thyroid nodules; (2) Description of classic and new features of thyroid nodules; (3) Identification of independent risk factors of thyroid cancer in 3D ultrasound data by multiple logistic regression analysis; (4) Analysis of feasibility of 3D ultrasound for application in thyroid cancer databases.

## Methods

The study was carried out in compliance with Helsinki Declaration. From years 2003-2005, 92 thyroid nodules in 82 patients referred for fine needle biopsy (FNB) were examined with 3D gray-scale and power Doppler sonography. Seventy-one thyroid nodules larger than 7 mm in 65 patients with established diagnosis (benign nodule or cancer) by FNB and/or pathology after surgery were evaluated retrospectively in 3D sonography volumes [Table 1]. The purpose and procedure was explained to the patients and their informed consent was obtained. Initially patients were prospectively evaluated using conventional sonography of the whole thyroid gland and the neck lymph nodes. In multinodular goiter, suspicious nodules were identified by the presence of any combination of the following criteria: dominant (the largest or enlarging) nodule, hypoechoic nodule, nodule with poorly defined borders, calcification/microcalcification-like echogenic foci (CAL), and increased central vasculature [12-15]. Thyroid nodules in patients with carcinoma established by FNB diagnosis before 3D sonography were also included in the study.

**Table 1:** General findings of 71 retrospectively analyzed thyroid nodules.

	Benign nodule	Cancer
Number of nodules	55	16

Nodules in multinodular goiter	45	14

Solitary nodules	10	2

Women	46	9

Men	4	6

Age of patients (years)	22-76	26-70

Final diagnoses were established by FNB and pathology after surgery for all 16 carcinomas (15 papillary cancers, 1 medullary cancer) and 12 benign nodules, and by multiple FNB and at least 2 years follow up for 43 benign nodules. FNB was guided by ultrasound imaging. The FNB diagnoses were organized into 4 categories - (1) inadequate material (unsatisfactory or nondiagnostic): smears with few or no follicular cells; (2) benign or negative: group including colloid nodule, Hashimoto's thyroiditis, cyst, thyroiditis; (3) suspicious or indeterminate: cytologic results that suggest a malignant lesion but do not completely fulfill the criteria for definitive diagnosis, including follicular neoplasms, Hürthle cell tumors and atypical papillary tumors; (4) malignant or positive: group consisting of primary thyroid cancers [16]. Nodules diagnosed with FNB a follicular neoplasm that were not subjected to surgery were excluded from the retrospective analysis.

Three-dimensional ultrasound studies were performed using a dedicated 4D sonographic scanner (Voluson 730; GE Medical Systems, Kretz Ultrasound, Zipf, Austria) equipped with an automatic linear 6-12 MHz 4D probe. All volumes were registered on a magneto-optical disk. The images were transferred to a personal computer and evaluated with 3D VIEW 2000 software (GE Medical Systems, Kretz Ultrasound, Zipf, Austria), which provides the same viewing interface as the scanner.

Gray-scale and power Doppler volumes were acquired. Power Doppler studies were performed with pulse repetition frequency (PRF) and color gain set just above the noise level (PRF = 0.9 kHz) and with the wall motion filter set to "low" to optimize for slow flow detection. Two radiologists blinded to the results of the FNB and pathology examinations independently reviewed the images.

Contiguous slices, from one border to the opposite border of the nodule were evaluated interactively from gray-scale volumes. The nodule shape in the plane parallel to the ultrasound probe surface (c-plane), echogenicity, margins of the nodule and presence of calcification/microcalcification-like echogenic foci (CAL) in the plane of the array of probe crystals (a-plane), were evaluated in multiplanar reformation (MPR) mode and with our original method of thin-slice volume rendering using a smooth surface algorithm [11].

**Figure 1:**
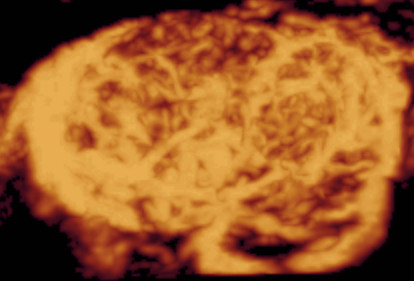
**Three-dimensional power Doppler ultrasound of Hürthle cell adenoma: presentation of the whole nodule**. On the 3D rendered image of the whole nodule the peripheral and central vessels overlap making the evaluation of the abundant vascularization difficult. (See also: Additional file 1).

**Figure 2:**
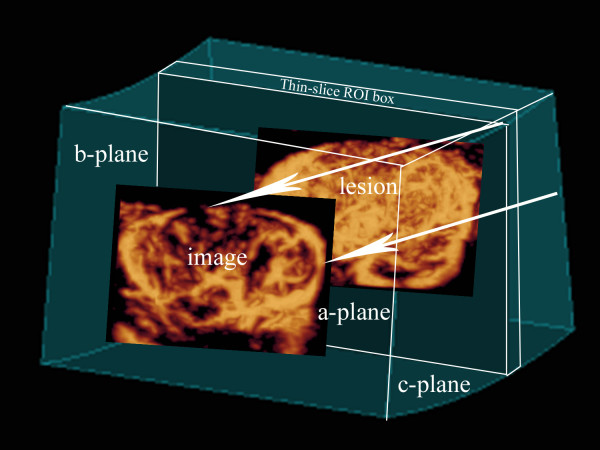
**Schematic presentation of the 3D sonographic method: thin-slice volume rendering of vessels visualized with power Doppler**. The Color max algorithm is applied to the thin-slice region of interest (ROI) box, with the resultant 2D image of the vessels in the a-plane.

**Figure 3:**
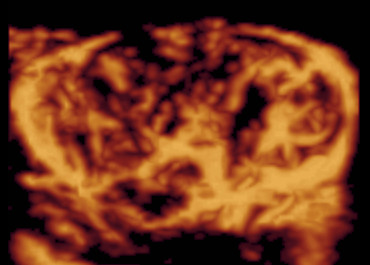
**Three-dimensional power Doppler ultrasound of Hürthle cell adenoma: presentation with thin-slice rendering method (the nodule from figure 1)**. Thin-slice rendering permits evaluation of the central and peripheral vessels (See also: Additional file 2).

In the evaluation of vessels on rendered 3D power Doppler volumes of whole nodules, the peripheral and central vessels overlapped and classification of the vascularization was difficult, especially in cases where it was abundant [Figure 1, Additional file 1]. Thus, we applied original thin-slice volume rendering of color data alone, employing the 100% color max algorithm [Figure 2, 3, Additional file 2]. The thickness of the slice was approximately 15-25% of the maximal nodule diameter. To define the location of nodule borders on a volume-rendered image, it was displayed on the same screen as the MPR presentation of gray-scale with color. The vessels were evaluated interactively with 360° rotation of the volume around the central axis of the nodule joining the anterior and posterior surfaces of the thyroid gland. The pattern of nodule vascularization, central vessel alignment, maximal extent of the peripheral vessel component and maximal area of the central vessel component were evaluated.

Statistical analysis was performed with Stata v. 8.2 (Stata Statistical Software: Release 8.2, Stata Corporation, College Station, TX, USA). The agreement between the observers and the gray-scale techniques (MPR and thin-slice rendering) was evaluated with κ statistics using the scale of Landis and Koch: κ < 0 denotes poor reproducibility, 0-0.20 slight, 0.21-0.40 fair, 0.41-0.60 moderate, 0.61-0.80 substantial, and 0.81-1.00 almost perfect reproducibility [17]. For evaluation of the independent risk factors of thyroid cancer identified by 3D ultrasound, multiple logistic regression analysis was applied. To assess the echogenicity of thyroid nodules, the Fisher exact test was applied. The significance threshold was set at 0.05.

## Results

### Gray-scale 3D ultrasound

A summary of features of nodules examined with 3D gray-scale ultrasound is presented in Table 2. The Fisher exact test indicated that the percentage of cancers is significantly higher in the group of hypoechoic and mixed echogenicity nodules (p = 0.015). The feature of ill-defined nodule borders was 63-69% sensitive and 82-85% specific in the case of thyroid cancer. The sensitivity and specificity of CAL in MPR presentation were respectively, 81-88% and 38-44%, and with thin-slice rendering, 88-94% and 22-25%. In the case of one cancer, thin-slice rendering revealed CAL that was not observed in MPR presentation. Analysis of the shape of the nodule in the c-plane was possible in 44-54% of cases in MPR presentation and in 100% of cases with thin-slice rendering. The analysis in the c-plane was therefore performed using thin-slice rendered images. A lobulated shape was 94-100% sensitive and 47-58% specific for thyroid cancer. The level of agreement between observers and between techniques is presented in Tables 3 and 4.

**Table 2:** Features of benign thyroid nodules and thyroid cancer in 3D gray-scale ultrasound [echogenicity, borders, calcification/microcalcification-like echogenic foci (CAL), shape in c-plane (parallel to ultrasound probe)] in multiplanar reformation mode (MPR) and by thin-slice rendering with surface algorithm (Surf).

		BENIGN *Observer 1*	BENIGN *Observer 2*	CANCER *Observer 1*	CANCER *Observer 2*
*ECHOGENICITY*:					
**Hypoechoic**	**MPR**	20	22	12	11
	**Surf**	20	21	11	12
**Mixed**	**MPR**	17	17	4	5
	**Surf**	22	20	5	4
**Isoechoic**	**MPR**	17	15		
	**Surf**	12	13		
**Hyperechoic**	**MPR**	1	1		
	**Surf**	1	1		

*BORDERS*:					
**Ill-defined**	**MPR**	8	10	10	11
	**Surf**	5	10	8	9
**Well-defined**	**MPR**	47	45	6	5
	**Surf**	50	45	8	7

*CALCIFICATION/MICROCALCIFICATION-LIKE ECHOGENIC FOCI*					
**Present**	**MPR**	31	34	14	13
	**Surf**	43	41	15	14
**Absent**	**MPR**	24	21	2	3
	**Surf**	12	14	1	2

*SHAPE IN C-PLANE*					
**Lobulated**	**Surf**	23	29	16	15
**Oval**	**Surf**	32	26	0	1

(mixed) echogenicity - hypo- and isoechogenic

**Table 3:** Observer agreement (κ statistics) over the evaluation of features of thyroid nodules in multiplanar reformation mode (MPR) and by thin-slice rendering with surface algorithm (Surf).

		κ	p
Echogenicity	MPR	0.70	< 0.0001

Echogenicity	Surf	0.71	< 0.0001

Borders	MPR	0.82	< 0.0001

Borders	Surf	0.60	< 0.0001

CAL	MPR	0.57	< 0.0001

CAL	Surf	0.44	= 0.0002

Shape in c-plane	Surf	0.57	< 0.0001

calcification/microcalcification-like echogenic foci (CAL); c-plane - plane parallel to ultrasound probe

**Table 4:** The agreement (κ statistics) between features of thyroid nodules evaluated in multiplanar reformation mode and by thin-slice rendering with surface algorithm.

	OBSERVER 1		OBSERVER 2	
	κ	p	κ	p
Echogenicity	0.85	< 0.0001	0.91	< 0.0001

Borders	0.80	< 0.0001	0.93	< 0.0001

CAL	0.56	< 0.0001	0.73	< 0.0001

calcification/microcalcification-like echogenic foci (CAL).

### Analysis of vascularization of thyroid nodules in 3D power Doppler ultrasound

Analysis of vascularization of benign thyroid nodules and thyroid cancers by thin-slice rendering of 3D power Doppler ultrasound is presented in Table 5. Agreement between observers in the evaluation of vascularization of thyroid nodules is presented in Table 6.

**Table 5:** Analysis of vascularization of benign thyroid nodules and thyroid cancers in 3D power Doppler ultrasound evaluated with thin-slice rendering.

		BENIGN *Observer 1*	BENIGN *Observer 2*	CANCER *Observer 1*	CANCER *Observer 2*
*PERIPHERAL VESSELS* *% of the circumference of the nodule*					
**0**	**0 - 10**	4	2	4	4
**1**	**11-25**	4	5	1	1
**2**	**26-50**	10	10	4	4
**3**	**51-75**	7	10	4	3
**4**	**76-100**	30	28	3	4

*CENTRAL VESSELS* *% of the area of the nodule*					
**0**	**0**	8	10	2	2
**1**	**1-25**	13	12	8	9
**2**	**26-50**	8	7	1	0
**3**	**51-75**	13	10	2	1
**4**	**76-100**	13	16	3	4

*ALIGNMENT OF CENTRAL VESSELS*:					
**Regular**		38	35	5	6
**Chaotic**		5	5	3	1
**Not possible to define**		12	15	8	9

**Table 6:** Observer agreement (κ statistics) over the evaluation of vascularization of thyroid nodules by 3D power Doppler ultrasound with thin-slice rendering.

	κ	P
Extent of peripheral vessels	0.93	< 0.0001

Area of central vessels	0.95	< 0.0001

Alignment of central vessels	0.72	< 0.0001

The pattern of vascularization of benign nodules was predominantly peripheral/central (seen in 85% of nodules), followed by a peripheral pattern (seen in 7-15% of nodules). No vessels were visible in 4-7% of nodules. Cancers presented predominantly peripheral/central vascularization (seen in 75% of cancers), followed by a central pattern (seen in 12.5% of cancers). No vessels were visible in 12.5% of cancers.

The regularity of central vessel alignment could not be evaluated in 50-56% of cancers.

**Figure 4:**
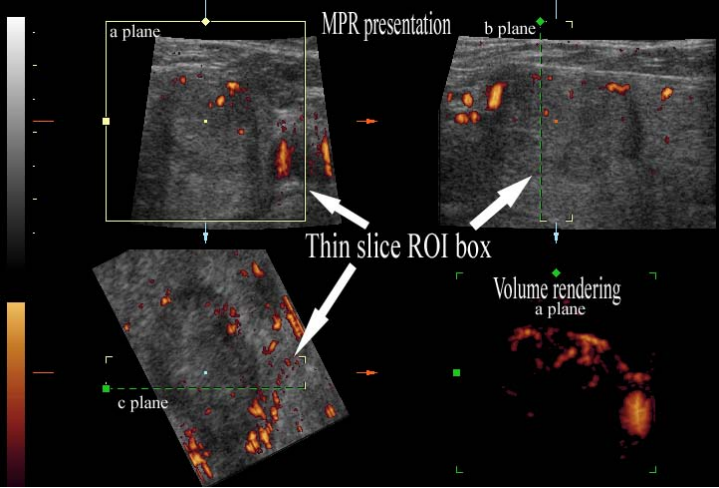
**Three-dimensional power Doppler ultrasound of papillary cancer with low density of vessels**. Multiplanar reformation (MPR) mode presentation and thin-slice volume rendering (lower right corner). The maximal density of the vessels was evaluated on the volume-rendered image (in the a-plane). The borders of the cancer on the a-plane image in MPR were correlated with the distribution of vessels on the volume-rendered image. The density of the vessels is in the lowest range: 1 = (1-25%) of area of the nodule; ROI - region of interest.

**Figure 5:**
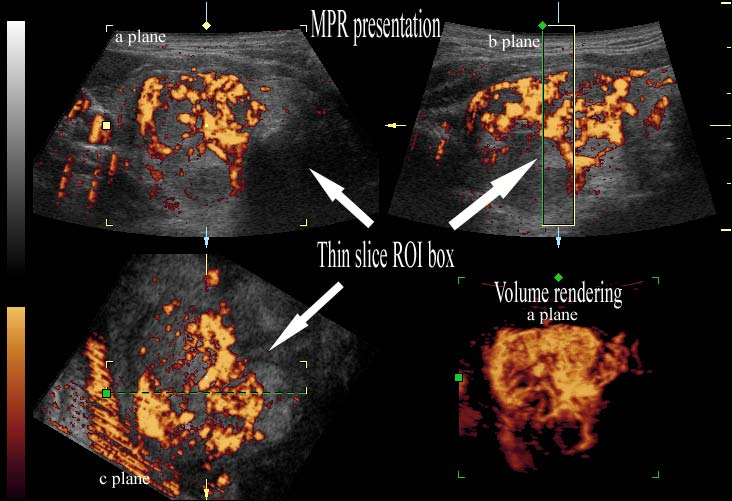
**Three-dimensional power Doppler ultrasound of papillary cancer with high density of vessels**. Multiplanar reformation (MPR) mode presentation and thin-slice volume rendering (lower right corner). The density of the vessels is in the highest range: 4 = (76-100%) of area of the nodule; ROI - region of interest.

A density of central vessels in ranges 1 and 4 [Figure 4, 5], correlated with an increased probability of cancer, described in logistic regression analysis (discussed below); this feature was 75-81% sensitive and 49-56% specific for a diagnosis of thyroid cancer.

### Multiple logistic regression analysis of 3D ultrasound of thyroid nodules

Multiple logistic regression analysis was applied for differential diagnosis of thyroid nodules [Table 7].

**Table 7:** Evaluation of 3D ultrasound features of thyroid cancer with multiple logistic regression analysis.

	OR	P
Ill-defined borders (a-plane, MPR mode)	8.3	0.005

Ill-defined borders (a-plane, thin-slice rendering)		> 0.1

Presence of calcification/microcalcification-like echogenic foci		> 0.1

Lobulated shape (c-plane, thin-slice rendering)	10	0.044

Extent of peripheral vessels		> 0.1

Central vessels: (1-25%) or (76-100%) of the area of the nodule	7.3	0.016

Alignment of central vessels		> 0.1

odds ratio (OR); a-plane - plane of the array of probe crystals; c-plane - plane parallel to ultrasound probe surface; multiplanar reformation mode (MPR); thin-slice rendering with surface algorithm.

**Figure 6:**
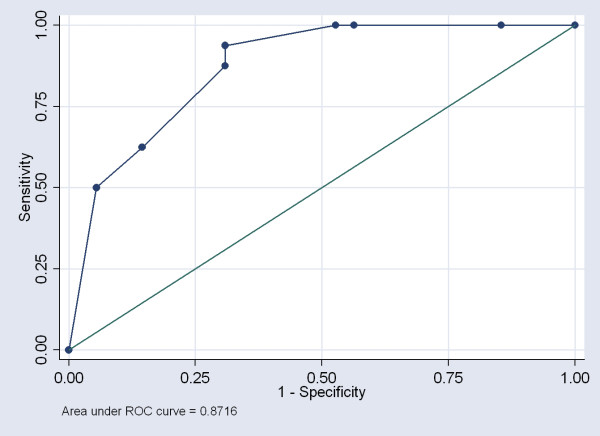
**The receiver operating characteristic (ROC) curve for multiple logistic regression analysis of 3D ultrasound features of thyroid cancers**. The model is highly predictive (area under ROC curve = 0.87).

The analysis revealed 3 statistically significant independent risk factors of thyroid cancer in 3D ultrasound data: ill-defined nodule borders on the a-plane in MPR mode; lobulated shape of the nodule in the c-plane on thin-slice rendering; and maximal area of the central vessel component within ranges 1 (1-25%) or 4 (76-100%). Our model of multiple logistic regression analysis proved to be highly predictive (area under ROC curve = 0.87) [Figure 6].

To optimize the detection of thyroid cancers, the sensitivity and specificity for combinations of parameters for hypoechoic or mixed echogenicity nodules displaying at least one risk factor were calculated. The best combination was for ill-defined borders in MPR mode or lobulated shape in the c-plane - sensitivity 100% and specificity 60-69%; the application of these criteria for referral to FNB would had decreased the number of biopsies from 71 to 38 without missing a malignant nodule.

## Discussion

The designation of thyroid nodules for FNB is especially important in multinodular goiter. This condition is often diagnosed by ultrasound examination, especially in the elderly and in people living in iodine-deficient areas. However, multinodular goiter can no longer be regarded as an indicator of benignity of thyroid nodules. Multiple studies have found a similar incidence of cancer in patients with a multinodular goiter and in those with a solitary nodule. In Poland over 50% of differentiated thyroid cancers are diagnosed in patients with multinodular goiter. In our study subjects, 14 of 16 cancers were localized in multinodular goiters. It is often impossible to subject all nodules in a multinodular goiter to FNB and this is why it is so important to use ultrasound examination to identify the nodules with suspicious features that require FNB [2,3,18-20].

So far, a few risk factors of thyroid cancer have been identified in studies using ultrasound. However, no single ultrasound feature has been identified that is 100% sensitive and specific for thyroid cancer. To increase the sensitivity and specificity, the use of combinations of different features has been proposed [21].

Gray-scale 3D ultrasound has recently been applied to the investigation of thyroid nodules [11]. The general advantages of three-dimensional ultrasound include following ones:

➢ Separation in time of image aquisition and analysis,

➢ Saving of examination room time,

➢ Less time on scanning - less injury problems,

➢ Diversyfication of sonographers work (scanning, reconstruction),

➢ Less operator dependent,

➢ Increase of confidence in diagnosis,

➢ Possibility of remote consultation,

➢ Precise evaluation of lesion diameters and volumes,

➢ Additional, unlimited planes of visualization,

➢ Unlimited combinations of rendering algorithms - new information,

➢ Presentation of data similar to CT or MR (TUI),

➢ Convenient presentation for clinician and for teaching,

➢ Possibility of inclusion in patients data bases with future evaluation of new features or even with new rendering algorithms.

**Figure 7:**
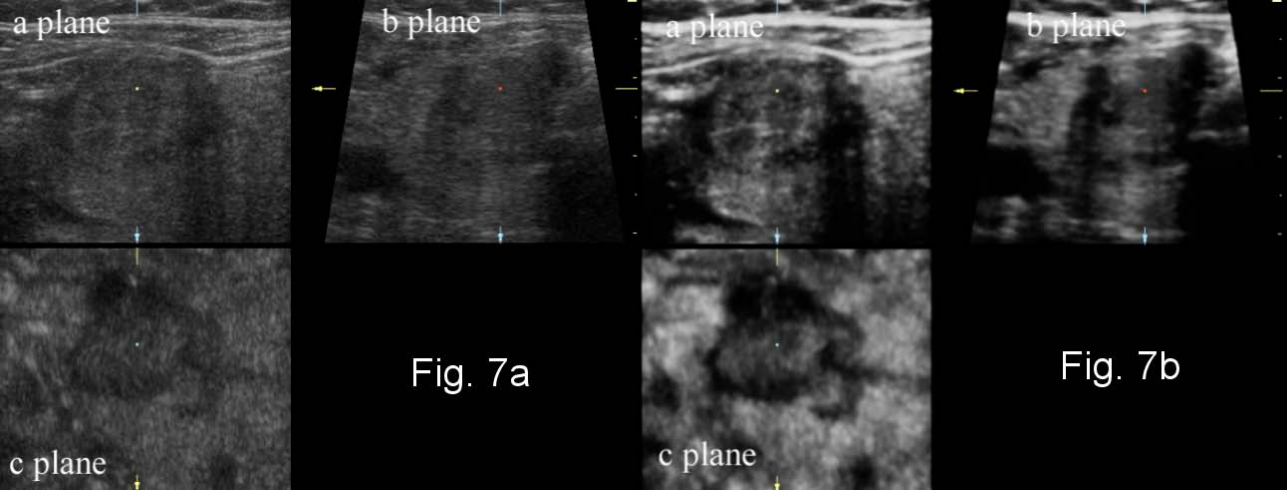
**Multiplanar reformation (MPR) presentation of three-dimensional gray-scale ultrasound of papillary thyroid cancer without and with static volume imaging mode**. (a) classic MPR presentation; (b) MPR presentation of the lesion with a new 3D postprocessing technique - static volume contrast imaging (VCI). Thin-slice volumes of 2 mm thickness with the surface rendering algorithm increases the contrast and reduces the noise on the resultant images.

**Figure 8:**
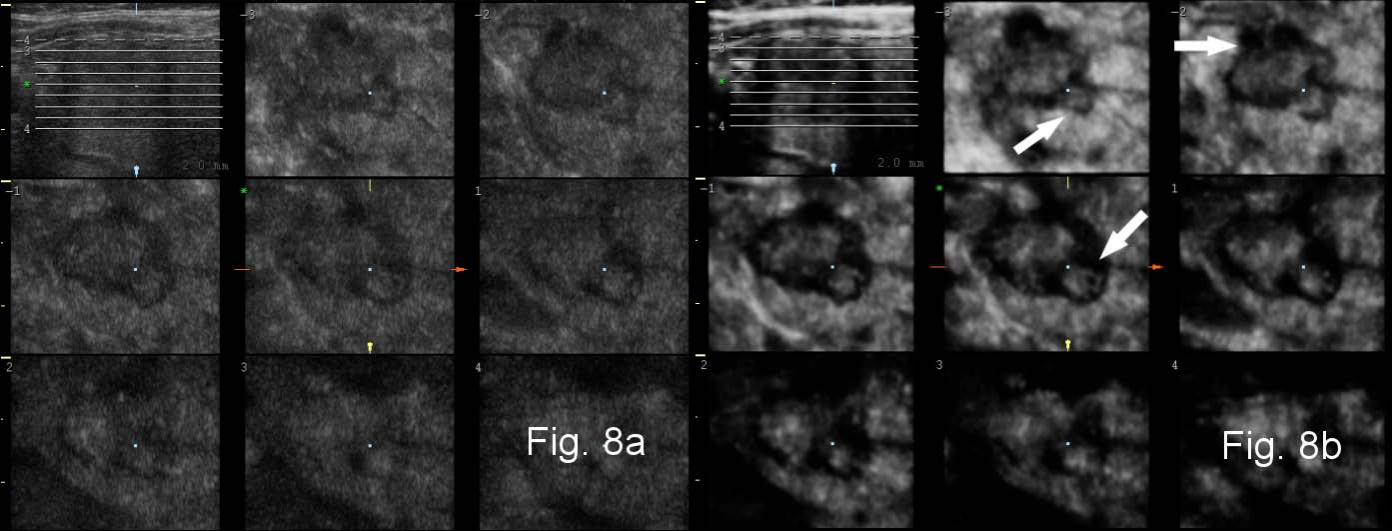
**Presentation of three-dimensional gray-scale ultrasound of papillary thyroid cancer with a new 3D postprocessing technique - tomographic ultrasound imaging (TUI)**. (a) a set of sequential slices 2 mm apart in the c-plane (plane parallel to the surface of the ultrasound probe) covering the whole nodule and localizer in the axial plane (upper left corner); (b) TUI of the whole nodule with static volume contrast imaging (VCI) - a set of c-plane images as a result of rendering of sequential thin slice volumes (2 mm thickness) with the surface algorithm and axial localizer (upper left corner). On coronal images the lobulations of the carcinoma (arrows) are more readily distinguished than in Fig. 8(a) due to improved contrast and reduced noise.

The present study describes thyroid cancer risk factors based on gray-scale and power Doppler 3D ultrasound. Three-dimensional ultrasound permits the evaluation of tissues by the application of different rendering algorithms in countless combinations. For the evaluation of volume data, we applied the original thin-slice method. Each gray-scale ultrasound volume was evaluated with a surface algorithm that enabled reduction of noise and speckles, and improved contrast. These features improved visualization of the lesions on images parallel to the ultrasound probe and were also more sensitive for high echoes of the calcification/microcalcification type. Due to characteristics of the software applied, the thickness of the evaluated slices was approximately 15-25% of the maximal diameter of the lesion. A new software tool called static Volume Contrast Imaging (VCI) (applied to some archived volumes in our study) enables the evaluation of the lesion with different combinations of rendering algorithms (including surface) with a fixed thin-slice thickness of as little as 2 mm [Figure 7]. In addition, a new option called Tomographic Ultrasound Imaging (TUI) allows the presentation of a series of slices (e.g. covering the whole lesion) in a similar way to magnetic resonance or computed tomography data [Figure 8]. Both VCI and TUI should greatly enhance and accelerate the evaluation of features of thyroid nodules with the thin-slice method.

In published 2D color or power Doppler studies of thyroid cancers, increased central vascularization with irregular alignment was found. However, the criteria used to evaluate central vascularization were not uniform, so it is difficult to compare the data [4,12,13,15].

The vascular net of the nodule may be visualized with 3D power Doppler ultrasound. However, due to the overlaying of peripheral and central vessels when the whole lesion was included in rendering (particularly confusing in highly vascularized nodules), we applied the original thin-slice method for the evaluation of vascularization. This permits the visualization of longer fragments of vessels rather than the cross-sections that may be seen with conventional power Doppler ultrasound. However, in at least half of thyroid cancers, the thin-slice method did not allow evaluation of the regularity of the central vessels; in most of these nodules, this could be related to the lowest density of central vessels and in some cases, to the highest density of central vessels.

In many nodules, central vascularization is not homogenous and the choice of the plane for evaluation and documentation in 2D ultrasound examination depends on the operator. In evaluations based on archived images from earlier 2D examination, alternative sections are not always available for inspection. In our study using 3D power Doppler ultrasound, the vessels of the whole nodule were evaluated during a 360° rotation of the volume around the antero-posterior axis and the section used to describe the vascularization was chosen by the radiologist. To increase the objectivity of the evaluation, a five point score describing the visually estimated percentage of the area of the nodule covered by central vessels was applied. To correlate the present study with previously published 2D studies that identified increased central vascularization in cancers, the image with maximal density of vessels was chosen for the rating. Central vessel densities in the lowest range (1-25% of nodule area) were found in over half of the cancers, despite optimization of power Doppler acquisition for slow flow and evaluation of vessels in slices with a thickness of 15-25% of the maximal diameter of the nodule. Thus, in contrast to many previous reports, poor central vascularization was frequently observed in thyroid cancers in this study. It is noteworthy that our material included many small cancers that may be hypovascular because of their high fibrous component [4,22].

There is usually some degree of observer variation in both the clinical evaluation and interpretation of imaging studies of thyroid disorders, and this must always be taken into account in decision-making. In previous 2D ultrasound examinations of thyroid nodules, poor reproducibility was reported in the evaluation of echogenicity, borders and volume; while good reproducibility was found in the evaluation of presence of calcifications, central vessels or cystic components [23-26]. However 3D ultrasound in the present study proved to deliver overall good interobserver reproducibility in evaluation of thyroid nodule features. The poorest agreement on evaluation of gray-scale features on 3D ultrasound was at least moderate (κ≥0.41). Least agreement between observers and between techniques occurred in the evaluation of CAL.

The evaluation of 3D power Doppler ultrasound proved to deliver even better interobserver reproducibility. The κ statistics for evaluation of vascularization revealed at least substantial agreement between observers (κ≥0.61).

The differences in reproducibility of certain features of nodules between previous 2D studies [25] and 3D ultrasound may be partly due to peculiarities of evaluation using the two techniques. With conventional ultrasound, often selected images of the nodule are evaluated retrospectively, whereas with 3D ultrasound, continuous images through the whole nodule are evaluated interactively.

We applied multiple logistic regression analysis to assess the risk factors of thyroid carcinoma identified by 3D ultrasound. Numerous features of thyroid nodules were investigated by gray-scale and power Doppler ultrasound. Our study revealed 3 independent risk factors of thyroid cancer:

✓ ill-defined nodule borders on MPR (sensitivity 63-69% and specificity 82-85%) - a feature known from 2D ultrasound studies,

✓ lobulated shape of the nodule in the plane parallel to the ultrasound probe (sensitivity 94-100% and specificity 47-58%) - a new feature specific to 3D ultrasound,

✓ density of central vascularization within the lowest or highest ranges (sensitivity 75-81% and specificity 49-56%) - the high percentage of cancers with a vessel density within the lowest range is a novel finding.

Ill-defined borders are an established risk factor of thyroid cancer. Moreover, ill-defined borders are a feature of aggressive behavior in papillary microcancer of the thyroid [12,27-29], which highlights the importance of investigating nodules presenting with an ill-defined border.

We analyzed the shape of thyroid nodules in the plane parallel to the ultrasound probe. A lobulated shape proved to be an independent predictor of thyroid cancer. In previous studies using conventional ultrasound, a lobulated shape was described as a feature of nodule borders. However, due to our combined observation of micro- and macrolobulated appearance, we established that this feature describes nodule shape. The disorganized tumor growth that produces the lobulated shape may be related to optimization of the supply of nutrients in an environment containing a heterogenous net of thyroid vessels [30]. The more frequent appearance of lobulated cancers in the plane parallel to the ultrasound probe (c-plane), in contrast to findings in 2D ultrasound studies [27,29], may be attributed to the arrangement of vessels in the thyroid gland and differences in the analysis of conventional ultrasound images compared to the analysis of 3D ultrasound volumes. Moreover, the plane parallel to ultrasound probe - the coronal plane in the case of thyroid examination - is characterized by the largest surface and provides the most space in the gland for the propagation of side lobes resulting in a lobulated shape.

There have been attempts to join individual volumes to obtain a volume encompassing the whole thyroid. Such an approach would enable physicians to fully archive the anatomy and pathology and to compare them during a follow-up [31].

Maybe in the future construction of three-dimensional probes with wider field of view (automatic 3D probes with wider footprint together with application of trapezoid field of view) would facilitate the acquisition of the volume covering the whole lobe or even the whole thyroid.

Even the new generation of elastography, called supersonic shear wave elastography [32,33] has already been equipped with three dimensional imaging capabilities at the end of year 2010. As in the real world, biological objects are three-dimensional, their three-dimensional analysis seems to be the most precise and desirable one.

According to Mitchell et al. an optimal management strategy for thyroid incidentalomas could be developed using an evidence-based approach in which systematic evaluation of obtained data is used. All physicians involved in the care of those with thyroid disease (radiologists, along with endocrinologists and surgeons) should be encouraged to submit data to national databases and participate in properly randomized studies to address the optimal management strategy in the treatment of incidentally detected thyroid nodules [34]. As high-resolution ultrasound is the most useful modality for imaging thyroid nodules, the databases should contain the most complete ultrasound documentation. Our study indicates that, the most appropriate technique for this purpose appears to be 3D ultrasound as it stores volumes describing the whole lesion or organ. Future detailed evaluations of the data are possible, looking for features that were not fully appreciated at the time of collection or applying new algorithms for volume rendering in order to glean important information.

## Conclusions

(1) Three-dimensional ultrasound is a practical and reproducible method for the evaluation of thyroid nodules. It enables precise, sonographic, volumetric evaluation of morphology of thyroid lesions as: echogenicity and shape of the lesion, its borders, calcification/microcalcification-like echogenic foci and vascularization;

(2) Three-dimensional ultrasound stores volumes comprising the whole lesion or organ. Future detailed evaluations of the data are possible, looking for features that were not fully appreciated at the time of collection or applying new algorithms for volume rendering in order to gain important information.

(3) Three-dimensional ultrasound data could be included in thyroid cancer databases.

(4) Further multicenter large scale studies are warranted.

## Competing interests

The authors declare that they have no competing interests.

## Authors' contributions

RZS conceived, designed, coordinated and evaluated the study, carried out the ultrasound examinations and wrote the manuscript, WSJ participated in design, coordination and evaluation of the study, JSS governed clinical part of the study: patient selection, evaluation, consultations and establishment of final diagnosis, KTS participated in the design and evaluation of the study. All authors have read and approved the final manuscript.

## Supplementary Material

Additional file 1 **Cine presentation of three-dimensional power Doppler ultrasound of Hürthle cell adenoma, including of the whole nodule**. On the 3D rendered image of the whole nodule the peripheral and central vessels overlap making the evaluation of vascularization difficult. (Case also presented on Figure 1). Click here for file

Additional file 2 **Cine presentation with thin-slice rendering method of tree-dimensional power Doppler ultrasound of Hürthle cell adenoma (the nodule from additional file **1**)**. Thin-slice rendering permits evaluation of the central and peripheral vessels. (Case also presented on Figure 3). Click here for file
